# Next-generation sequencing improves pathogen identification in odontogenic abscesses, could this affect clinical outcomes?

**DOI:** 10.1007/s00784-025-06504-0

**Published:** 2025-09-02

**Authors:** Felix Benjamin Warwas, Felix Thol, Martin A. Sieber, Nikolai Spuck, Franz-Josef Kramer, Nils Heim

**Affiliations:** 1 Department of Oral and Maxillofacial Plastic Surgery, University Medical Center of the University Bonn, Venusberg Campus 1, Building 11, 2. OG, 53127 Bonn, Germany; 2 Institute of Medical Biometry, Informatics and Epidemiology, Medical Faculty, University of Bonn, Venusberg Campus 1, Building 11, D-53127 Bonn, Germany; 3 Institute for Functional Gene Analytics (IFGA), University of Applied Sciences Bonn-Rhein-Sieg, Rheinbach, Germany

**Keywords:** Odontogenic abscesses, Microbiome, Next-generation sequencing, NGS, Antibiotic resistance

## Abstract

**Objectives:**

This study aimed to evaluate the diagnostic performance of gene-based bioinformatic analysis via next-generation sequencing (NGS) compared to conventional culture and microscopy in identifying microorganisms and antibiotic resistances in odontogenic abscesses. Additionally, the influence of selected clinical indicators including prior antibiotic therapy, in-hospital antibiotic treatment, and detection of antibiotic resistance on length of stay (LOS) was analysed.

**Materials and methods:**

In patients undergoing extraoral incision and drainage of odontogenic abscesses at the Department of Oral and Maxillofacial Plastic Surgery, University Hospital Bonn, two microbiological swabs (NGS and conventional) were taken intraoperatively from each patient. Microbial profiles and resistance patterns were assessed and correlated with clinical data.

**Results:**

Bacteria were identified in 100% of NGS swabs compared to 68.1% in conventional swabs (*p* < 0.001). NGS detected a median of 8 bacterial genera per sample versus 1 via conventional methods, primarily due to superior detection of anaerobes (median 7 vs. 0). Both methods equally identified aerobic genera (median 1 each). Fungal organisms and antibiotic resistances were also more frequently detected using NGS (*p* < 0.001). Prior antibiotic therapy was associated with a significantly reduced LOS (*p* = 0.030). Neither the type of in-hospital antibiotic regimen nor the presence of resistance influenced LOS.

**Conclusions:**

NGS substantially enhances the detection of polymicrobial communities, including anaerobes and fungi, and identifies antibiotic resistances more effectively than conventional diagnostics in odontogenic abscesses.

**Clinical Relevance:**

NGS offers rapid, comprehensive pathogen profiling and resistance testing, supporting quick establishment of tailored antimicrobial therapy.

## Introduction

Odontogenic abscesses originate from the teeth or the periodontium and commonly arise from dental caries, apical periodontitis or after tooth extractions [1].

These infections account for a significant percentage of the patients in oral and maxillofacial plastic surgeries and clinics and are associated with high costs [2–4]. For the purpose of diagnosis and treatment, a classification into localised odontogenic abscesses and abscesses with a tendency to spread should be used [5]. Thus, the latter can lead to life-threatening localised (sinusitis, orbital cellulitis, mediastinitis) or systemic complications (sepsis) which are associated with high mortality rates [6, 7].

The triggering bacterial spectrum of odontogenic abscesses stems from the oral microbiome. Polymicrobial infections that are dominated by anaerobic microorganisms are therefore most typical [8].

Treatment of abscesses with a tendency to spread involves extraoral surgical drainage as well as intravenous antibiotics. In accordance with the current guidelines a microbiological swab should be taken during this procedure, which is then subjected to a conventional microbiological examination using microscopy and culture diagnostics [5]. Unfortunately, conventional microbiological analysis take several days to provide results, thus having seldomly real impact on clinical antibiotic therapy in odontogenic infections. Moreover, in view of the triggering bacterial spectrum, it is not surprising that in many cases no microbial evidence can be provided in everyday clinical practice, or only commensal bacteria can be identified [9].

The underlying cause is the cultivation of anaerobic microorganisms, which is associated with a number of pre-analytical and analytical errors [10, 11].

In result, calculated antibiotic therapy is predominantly used for odontogenic abscesses. However, as targeted antibiotic therapy is one of the most important pillars in the treatment of odontogenic infections, there is an urgent need for improvement in microbiological diagnostics.

In view of the increasing availability, decreasing costs and short analysis times, gene-based microbiological analysis offer a valid alternative [12].

This study aimed to investigate whether gene-based microbiological diagnostics using next-generation sequencing (NGS) can achieve an improvement in pathogen and resistance detection compared to conventional microbiological diagnostics using culture and microscopy. In addition, the effects of different antibiotic therapies and the detection of antibiotic resistance on treatment outcomes was investigated.

## Methods

### Patients

In the period from January 2022 until April 2023 deep wound swabs were collected from 51 patients undergoing treatment for an odontogenic abscess in the Department of Oral and Maxillofacial Plastic Surgery at the University Hospital Bonn. All patients received two swabs, from which one was analysed using NGS and the other using conventional microbiological analysis.

The study included patients suffering from an abscess originating from the mandibular teeth with a tendency to spread beyond their primary space of infection. All patients therefore underwent extraoral abscess incision via a submandibular approach. After sharp dissection down to the platysma, a blunt dissection down to the mandible was carried out. This was followed by subperiosteal preparation and collection of the swab, insertion of at least one drain and securing with sutures. In accordance with the current German guidelines, all patients received intravenous antibiotic therapy due to the diagnosis of compartment-abscess with a tendency to spread. In most cases, the first intravenous antibiotic was administered intraoperatively immediately after the swab collection. Oral antibiotic therapy was taken by approximately one third of the patients before hospitalisation. As part of the inpatient stay, standard antibiotic therapy with 3 g IV ampicillin/sulbactam tid was administered. In patients with penicillin allergy, the regimen was changed to 600 mg IV clindamycin tid, while 4/0.5g IV piperacillin/tazobactamen tid was used in patient with septic shock or septicaemia. Antibiotic therapy was continued until clinical symptoms had completely subsided.

The sequencable wound swabs were sent to Zymo-Research Europe GmbH, based in Freiburg, Germany (Mülhauser Str. 9, 79110 Freiburg im Breisgau) for gene-based microbiological analysis after sampling. Swabs for the conventional culture-based microbiological analysis remained at the University Hospital Bonn.

Written informed consent was obtained from each patient. This study conducted to the requirements of the Declaration of Helsinki from 1964 and was approved by the responsible ethics committee of the University of Bonn (reference 447/21).

### Methods

Using a curated reference database, the genetic material previously identified in the 16 s RNA analysis was assigned to organism profiles in a bioinformatic analysis (PrecisionBIOME™ project). The samples were analysed in several steps, which are explained in the following:

- *Sample collection*: The manufacturer Zymo-Research Europe GmbH (Mülhauser Str. 9, 79110 Freiburg im Breisgau, Germany) provides a sterile sampling kit containing a nucleic acid-stabilising solution called DNA/RNA ShieldTM. This solution can stabilise the microbial DNA present in the sample for up to 30 days at room temperature. After sampling, the swabs were dispatched for analysis.
- *DNA extraction, amplification and sequencing*: In a first step, the microbial DNA is extracted from the sample. The ZymoBIOMICS DNA Miniprep Kit is used for this according to the manufacturer's instructions (Zymo-Research Europe GmbH). The extracted DNA is then prepared for NGS analysis. This step includes library preparation using the Quick-16STM NGS Library Prep Kit (Zymo-Research Europe GmbH) as well as sequencing of barcoded amplicons using the MiSeq sequencing platform (Illumina, San Diego, CA). A separate kit was used to determine the absolute abundance of microorganisms (Femto Bacterial and Fungal DNA Quantification Kits; Zymo-Research Europe GmbH).
- *Bioinformatic analysis and taxonomic assignment*: Bioinformatic analysis was performed using PrecisionBIOMETM bioinformatics pipeline. Uclust was used to carry out the taxonomic classifications. This was done using PrecisionBIOMETM's own database. Phylotypes were computed as percentage proportions based on the total number of sequences in each sample. Absolute microbial quantification was performed using a real-time PCR approach. Here, primers targeting the V1-V3 and ITS regions were used for the quantification of bacteria and fungi, respectively.

The identification of antibiotic resistance genes was carried out using a proprietary sequencing method (Zymo-Research Europe GmbH). This involved an amplicon-based sequencing approach in which PCR primers were used to analyse the contained genetic material for at least eighty resistance genes.

In summary, this enables the taxonomic determination of all microorganisms and the identification of the antibiotic resistance genes present in the sample. Assignment of the genetic material is thereby possible down to the species level; for reasons of clarity, the genus level was used for the comparative analysis.

Conventional microbiological diagnostics and taxonomic determination were performed by using culture and microscopy diagnostic. The results of the conventional smears represent real world data from our clinic. Conventional analysis usually begins with culture on casein soya peptone blood agar, MacConkey agar and Schaedler-KV agar. For tests on fungi, Candiselect and Sabouraud agar is also used. An enrichment bouillon (brain–heart infusion and thioglycollate bouillon) is usually also prepared.

The aerobic agar media are analysed after 24 and 48 (or 72 in the case of filamentous fungi) hours, the anaerobic cultures after 72 h. The enrichment bouillons are checked daily for turbidity and incubated for 14 days; if there are signs of growth, they are examined under the microscope and seeded onto suitable culture media.

The identification of cultivated microorganisms is supported by Matrix Assisted Laser Desorption Ionisation Time of Flight Mass Spectrometry (MALDI TOF MS, Biomerieux).

Antibiotic susceptibility testing was performed according to the guidelines of the European Committee on Antimicrobial Susceptibility Testing (EUCAST) using Vitek2 (Biomerieux) for aerobic and facultative anaerobic bacteria, microbouillon dilution (Bruker) for strictly anaerobic bacteria and, in individual cases, by gradient diffusion test (E-test) or agar diffusion test. The selection of antibiotics and interpretation of resistance was based on EUCAST recommendations relevant to oral and maxillofacial infections. Due to space limitations and the standardization provided by EUCAST, detailed antibiotic types and concentrations are not listed individually [13].

The results of both analysis methods were therefore available for comparison. As 4 conventional samples were lost during the transport process, these were excluded from the comparative analysis. To enable better categorisation of the cohort and assessment of the treatment results, data from our clinical systems was analysed. Data such as the length of stay (LOS), antibiotic therapy used in hospital and abscess classification were collected. Patient data was handled according to the data protection law of germany regarding critical health care data (DSGVO).

### Statistical analysis

After completing the data collection, statistical analysis was performed using R version 4.3.1 (R Core Team, 2021, Vienna, Austria).

In the first part of the analysis, the conventional and the NGS swab were compared. To investigate the difference in cell count between patients for whom bacteria were detected in the conventional swab and patients for whom no bacteria were detected in the conventional swab, a Wilcoxon-Mann–Whitney test was applied. Comparisons of the detected genera (overall, anaerob, and aerob) between the conventional and the NGS swabs were performed based on the Wilcoxon signed-rank test. In addition, the two types of swabs were compared in terms of whether any bacteria, fungi and antibiotic resistances were detected using McNemar tests.

The second part of the analysis focused on LOS and investigated whether it is affected by certain patient characteristics, namely, previous antibiotic treatment (yes or no), type of antibiotic treatment in hospital (ampicillin/sulbactam, clindamycin or piperacillin/tazobactamen), whether at least one resistance was detected in the conventional swab (yes or no), and whether at least one resistance was detected in the NGS swab (yes or no). To this end, a time-to-event model can be applied, where the event of interest is discharge from hospital and time is measured in days. Here, univariable semiparametric logistic discrete hazard models were applied, which are common tools for the analysis of discrete time-to-event data. In these models, the so-called discrete hazard function

$$\uplambda \left(\text{t}\right)=P\left(T=t\right|T\ge t, x),$$

where $T$ is the time that the event of interest occurs and $x$ denotes a possible infulential variable of interest. That is, the conditional probability that the event of interest occurs at time point *t*, given that the event has not occurred up until that time point *t*, is estimated. The discrete hazard function is based on a binary variable that indicates whether an event occurred at time $t$ or not and, therefore, regression approaches for binary outcomes can be applied. In particular, a logistic regression model, where the effect of days since admission was modelled in a flexible way using a smooth function and the effects of the patient characterises (previous antibiotic treatment, type of antibiotic treatment in hospital, detection of at least one resistance in the conventional swab, and detection of at least one resistance in the NGS swab, respectively) were included as binary variables, was fitted to the data. Logistic discrete hazard models are appropriate here as they facilitate handling the skewed distribution the LOS and allow to account for the discrete scale that the LOS are measured on ($t=1, \dots , 20$ days). Based on these models, survival curves showing the probability of still being hospitalised dependent on the days since admission were determined. For more details on discrete time-to-event analysis, see Tutz and Schmid (2016) [14] and Berger and Schmid (2018) [15].

## Results

### Baseline characteristics

The median age at the swab collection and abscess incision was 57.8 years (interquartile range (IQR): 37.8–73.1). Slightly more than half of the cohort were men (52.9%). When admitted to hospital, 9.8% of the patients stated that they were allergic to antibiotics and 31.4% of the patients had already received oral antibiotic therapy. Approximately two thirds of the patients suffered from a perimandibular (37.3%) or submandibular (39.3%) abscess. These abscess locations were therefore the most common, while pterygomandibular, massetericomandibular or submental locations occurred less frequently (Table 1). In the majority of cases, the dental focus was on the molar teeth (Table 2).

**Table 1:** Baseline characteristics and frequency of different abscess localisations. Continuous variables are expressed as medians with interquartile ranges in brackets, categorical variables as absolute and relative frequencies (%). (BMI: Body Mass Index; kg: kilograms; m2: square meters)

Baseline characteristics	Patients
Age at operation (years)	57.8 (37.8; 73.1)
Male gender Smoking Allergies to Antibiotics	27 (52.9%) 18 (35.3%) 5 (9.8%)
Abscess localisation	
Perimandibular Submandibular Pterygomandibular Submental Massertericomandibular Other Previous antibiotic treatment	19 (37.3%) 20 (39.3%) 6 (11.8%) 3 (5.9%) 2 (3.9%) 1 (2%) 16 (31.4%)
BMI (kg/m2)	28.1 (24.1; 31.2)

**Table 2:** Dental focus of the abscesses (FDI tooth designation). Molar teeth of the lower jaw were the most common cause. Categorical variables are expressed as absolute and relative frequencies (%)

Teeth	Patients
34 36 37 38 43 44 45 46 47 48	3 (5.9%) 5 (9.8%) 9 (17.6%) 4 (7.8%) 1 (2%) 1 (2%) 5 (9.8%) 5 (9.8%) 6 (11.8%) 6 (11.8%)

### Microbiological analysis

Conventional microbiological smears were able to detect bacteria in 68.1% (32/47) of the cases. The gene-based microbiological analysis using NGS was able to detect bacteria in all swabs, which represents a significant difference (*p* < 0.001).

**Fig. 1:**
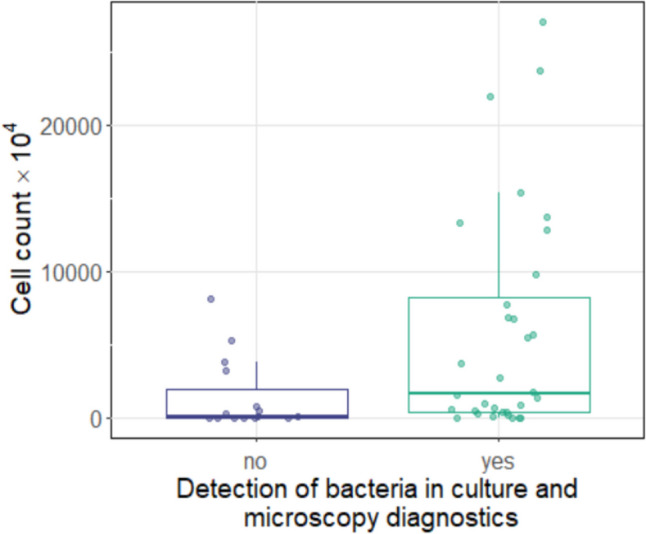
Boxplot showing the absolute frequency of bacterial cells detected (cell count) depending on pathogen detection in culture and microscopy diagnostics. Patients who had a pathogen detected by culture and microscopy had a higher median cell count (right boxplot). The difference was statistically significant (*p* = 0.006)

In cases where the conventional smear could not detect any bacteria, the median number of bacterial cells (cell count) in the NGS smear was reduced (8.99 × 10^5^, IQR: 5.40 × 10^4^–1.98 × 10^7^) compared to cases, where the conventional smear detected bacteria (1.66 × 10^7^, IQR: 3.70 × 10^6^–8.25 × 10^7^; *p* = 0.006; Fig. 1).

**Fig. 2:**
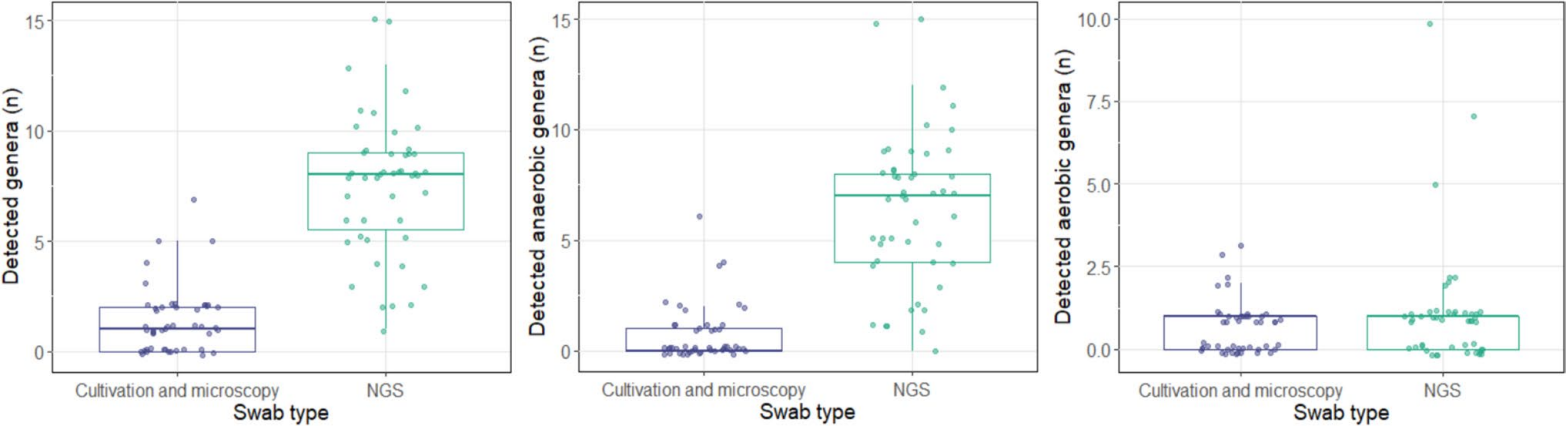
Boxplots showing the number of bacterial genera detected depending on oxygen tolerance in culture/microscopy diagnostics and analysis by NGS. On the left side culture/microscopy diagnostics, on the right side NGS (4a) total number of bacteria detected; (4b) anaerobic bacteria; (4c) aerobic bacteria. The number of bacterial genera detected differed significantly for the total number of bacteria (*p* < 0.001) and for anaerobic bacteria (*p* < 0.001), but not for aerobic bacteria (*p* = 0.365)

The total number of detected genera per smear also differed significantly between the conventional and NGS swab (*p* < 0.001). In the conventional smear, a median of 1 (IQR: 0–2) bacterial genus per smear was detected, while the gene-based analysis detected a median of 8 (IQR: 5.5–9) genera per smear (Fig. 2a). Considering the identification of the genera separately according to their oxygen tolerance, the gene-based swab detected more genera with a median of 7 (IQR: 4–8) compared to 0 (IQR: 0–1) for the conventional swab, regarding anaerobic bacteria (*p* < 0.001; Fig. 2b). There was no significant difference between the NGS smear with a median of 1 genus (IQR: 0–1) and the conventional smear with a median of 1 genus (IQR: 0–1) with regard to the detection of aerobic bacteria (*p* = 0.365; Fig. 2c).

**Fig. 3:**
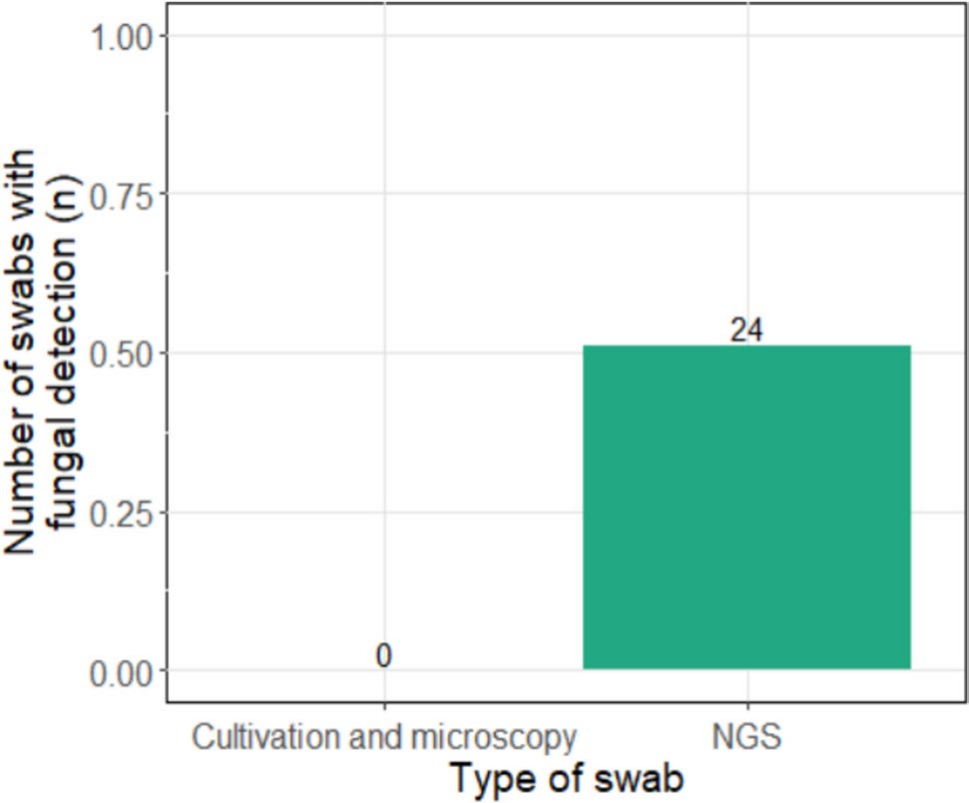
Comparison of fungal detection between the two swab types. On the left side culture/microscopy diagnostics, on the right side NGS. A difference was found between the two swab types (*p* < 0.001)

There was also a difference in the detection of other microorganisms. No fungi were detected in any of the conventional smears, while fungi could be detected in more than half of the patients (51.1%) in the NGS smear (*p* < 0.001; Fig. 3).

**Fig. 4:**
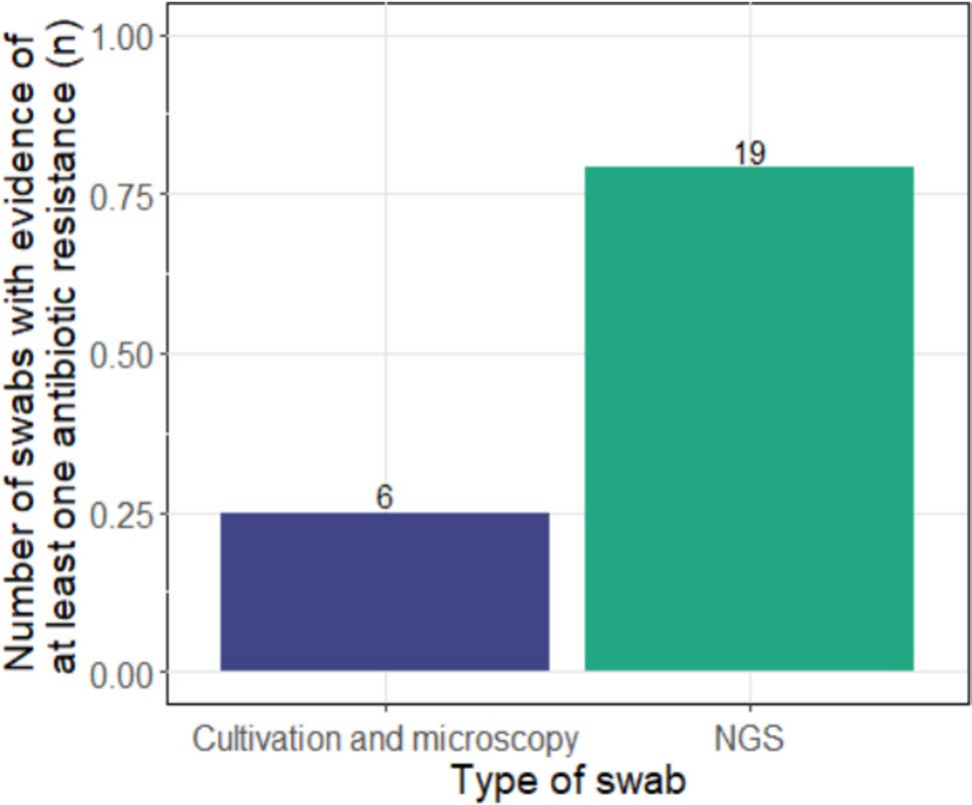
Comparison of the detection of antibiotic resistance in the two swab types. On the left the swab analysed by culture/microscopy diagnostics, on the right the NGS swab. A difference was found between the two swab types (*p* < 0.001)

In the detection of antibiotic resistance, the gene-based method was also able to detect resistance genes in more patients, than the conventional technique (*p* < 0.001). The NGS swab detected antibiotic resistance in more than twice as many swabs as culture-based diagnostics (Fig. 4).

### Treatment outcome

**Fig. 5:**
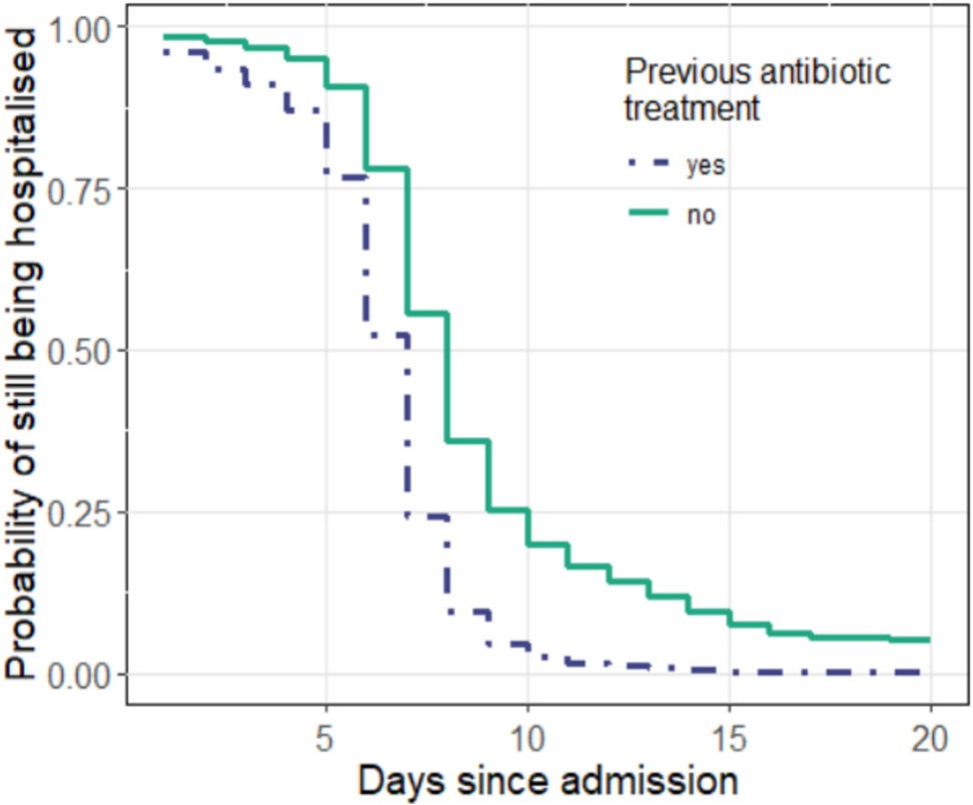
Duration of hospitalisation depending on previous oral antibiotic therapy. Patients who had received oral antibiotic therapy (blue) were hospitalised for a shorter period of time than the group without prior therapy (green). The difference was statistically significant (*p* = 0.030). The median time to discharge was seven and eight days, respectively

**Fig. 6:**
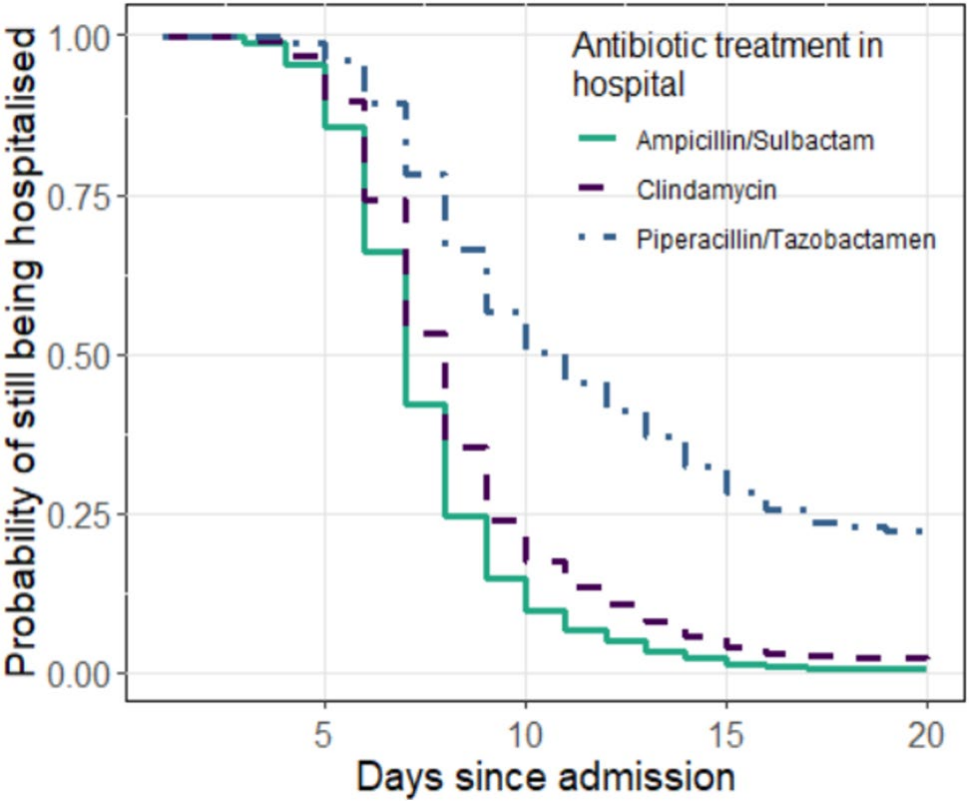
Duration of hospitalisation depending on antibiotic therapy during inpatient stay. Patients who had received antibiotic therapy with clindamycin (purple) or piperacillin/tazobactamen (blue) were hospitalised longer compared to the patients who had received standard therapy with ampicillin/sulbactam (green). No significant difference between the treatment groups was shown (*p* = 0.109). The median time to discharge was seven, eight and eleven days, respectively

The median duration of hospitalisation across all patients was seven days (Table 3). Patients who had received oral antibiotic therapy prior to admission were shown to have a higher probability of being discharged early (*p* = 0.030; Fig. 5). With regard to the antibiotic treatment administered in hospital, no effect on the probability of discharge from hospital was shown (*p* = 0.109). However, the survival curves in Fig. 6 suggest that patients who had not received standard therapy with ampicillin/sulbactam have a longer hospital stay. Patients who had received ampicillin/sulbactam were hospitalised for a median of seven days, while patients who had received clindamycin or piperacillin/tazobactamen were hospitalised for a median of eight and eleven days, respectively.

**Table 3:** Treatment outcomes. Continuous variables are expressed as medians with interquartile ranges in brackets, categorical variables as absolute and relative frequencies (%)

Outcome	Patients
Duration of Hospital Stay (Days)	7 (6;9)
Antibiotic Treatment in Hospital	
Ampicillin/Sulbactam Clindamycin Piperacillin/Tazobactamen	36 (70.6%) 8 (15.7%) 3 (5.9%)

**Fig. 7:**
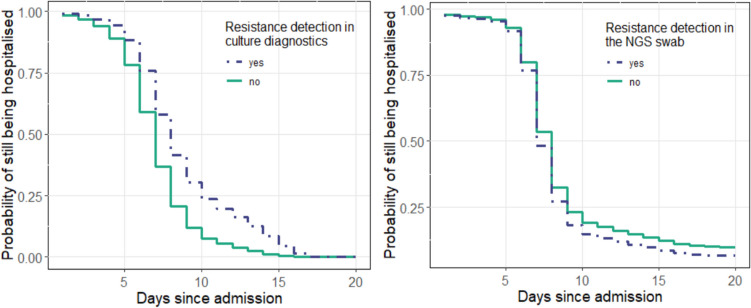
**a** Duration of hospitalisation depending on the detection of resistance in culture/microscopy diagnostics. Blue: Detection of at least one resistance. The difference was not statistically significant (*p* = 0.072). The median time to discharge was seven (green) or eight (blue) days. **b** Duration of hospitalisation depending on the detection of resistance in the NGS swab. Blue: Detection of at least one resistance. The difference was not statistically significant (*p* = 0.785). The median time to discharge was seven (blue) and eight (green) days respectively

Patients with evidence of clinical antibiotic resistance in the conventional swab tended to have prolonged hospitalisation but no effect was confirmed (*p* = 0.072; Fig. 7a). Such a tendency was not observed in patients in which resistance genes were detected using NGS (*p* = 0.785; Fig. 7b).

## Discussion

Dental or periodontal structures are one of the most common origins of infections in the head and neck area [16]. The treatment of odontogenic abscesses is therefore an essential part of the everyday clinical practice of oral and maxillofacial plastic surgeons [17, 18].

Life-threatening complications such as necrotising fasciitis, mediastinitis or sepsis, which are associated with high mortality rates of 10–40%, further emphasise the clinical relevance of this infections [7].

In the treatment of abscesses with a tendency to spread, surgical therapy is the main treatment according to the maxim „ubi pus, ibis evacua “. Antibiotic treatment still has a major role, since further spread and septicemia may be prevented by supplementary antibiotic therapy with a fitting calculated or, even better, targeted antibiotic. Changing antibiotics from a broader towards a targeted spectrum reduces the development of resistances and improves outcome by overcoming preexisting resistances.

Thus, only the combination of surgical drainage with targeted antibiotic therapy leads to adequate germ reduction [5]. While there is no question about the correct way of surgical drainage, changing calculated to targeted antibiotic therapy is only rarely performed. This is due to the fact, that conventional microbiological diagnostics takes several days to provide results, while having particular problems with the identification of anaerobic bacteria [10].

This situation is evident in other disciplines as well. Bioinformatic analysis methods such as ‘Maldi TOV’ have become established in everyday clinical practice in the diagnosis of septicemia. Due to the high accuracy and speed of this procedure, treatment outcomes can be improved through early pathogen identification and targeted antibiotic therapy [19].

Bioinformatic analysis based on the genetic material identified by NGS offers possibilities comparable to „Maldi TOV“. Therefore, this study aimed to compare NGS with the current conventional standard microbiological diagnostics of odontogenic abscesses. Furthermore, we wanted to examine how the used antibiotic therapy and the resistance behavior of germs affects the outcome of patients with odontogenic infections.

While NGS offers clear advantages in sensitivity and comprehensiveness, cost remains a limiting factor for routine clinical implementation. Conventional microbiological analysis is significantly less expensive, whereas NGS currently involves higher per-sample costs due to sequencing reagents, bioinformatic processing, and outsourced analysis. However, costs are steadily decreasing as the technology matures, and the potential for improved patient outcomes and reduced LOS may offset higher diagnostic expenditures in the long term.

The structure of our cohort was comparable with other studies in terms of age, gender distribution, abscess localisation and dental focus which ensures good transferability of the results.

As part of the quantitative analysis of the NGS swab, the absolute cell count was lower in patients in whom no bacteria could be detected in the conventional swab. If a hypothetical threshold value of microorganisms required for cultivation is not reached, germ detection is not achieved with conventional analysis. Thereby, the increased sensitivity of the gene-based analysis makes detection possible. A comparison of the two swab shows that gene-based diagnostics are superior, particularly in the detection of anaerobic bacteria, which emphasises the difficulties of conventional diagnostics for anaerobic bacteria. For the cultivation of bacteria, living organisms are required. Unfortunately there is an almost unavoidable contact with oxygen after the swab collection and during the pre-analytical phase. Hence many bacteria perish, which leads to a lack of detection. When detecting aerobic bacteria, both wound swabs therefore performed similarly, confirming the anaerobic gap for conventional analysis. The same could probably apply to the detection of fungi, as their cultivation is also associated with various pitfalls. The detection of antibiotic resistance by means of an inhibiting areola analysis also requires living organisms for cultivation. Without cultivation of bacteria, conventional resistance testing is not possible. In addition to the problems of detecting anaerobic germs, antibiotic testing is therefore impossible in many cases. This is not necessary for the detection of resistance genes using the gene-based analysis, which could explain the superiority of the NGS swab.

In addition to a comparison of diagnostic options, this study was intended to investigate the effects of both antibiotic treatment and the resistance situation on the LOS. In this regard, different studies have already shown that certain indicators such as blood values (C-reactive protein, white blood count) have an influence on the LOS [20–24]. In terms of anti-infective therapy, we were able to confirm that pre-hospital antibiotic therapy leads to a shorter hospital stay [25]. This might indicate that in most cases an effective antibiotic therapy is prescribed by the outpatient colleagues.

Furthermore, we were able to show that a deviation from the standard therapy with amoxicillin/clavulanic acid in favor of clindamycin or piperacillin/tazobactam tends to prolong the LOS. This could be due to a poorer resistance situation to clindamycin, which we use in cases of penicillin allergy [26–28]. Patients who suffered from more severe infections or who showed resistance in the conventional swab received piperacillin/tazobactamen. Both, a more pronounced infection and a higher incidence of antibiotic resistances can explain the prolonged LOS.

The fact that the time until the use of the correct antibiotic plays a role could also explain that patients with a resistance detected in the conventional smear showed a tendency towards prolonged hospitalisation, as a possibly less effective therapy was used up to this point. The LOS was not influenced by resistance gene detection using NGS in this study. This is explained by the fact, that in this study, the NGS analysis was not performed in a nearby lab, resulting in a delay of the results. Hence NGS results were not, as possible in another setting, able to influence clinical treatment. However, in a real-time hospital-based setting, NGS workflows can deliver results within 24–48 h, which is even faster than conventional culturing for anaerobic bacteria. This could enable earlier adjustments in antibiotic therapy, potentially improving outcomes and reducing hospitalisation times.

The detection of a resistance gene is not synonymous with resistance detected in culture diagnostics. Thus, an existing resistance gene does not necessarily manifest itself in clinical antibiotic resistance, which points to the greatest limitation of gene-based microbiological diagnostics and this study. Regarding the other limitations of this study, the small sample size and its monocentric design must also be mentioned.

Data from our study suggest that gene-based microbiological analysis is superior to conventional analysis in the detection of microorganisms and antibiotic resistance in odontogenic infections. However, it remains to be seen whether this advantage translates into improved outcomes for patients in a real-world setting.

We were able to show that the selection of an antibiotic therapy tailored to the triggering germ spectrum influences the treatment results of patients. Thus, patients with antibiotic-resistant germs that required broader antibiotic agents remained hospitalised longer, which can be explained by a delay caused by conventional antibiotic resistance testing. Whether this situation could be improved by a gene-based microbiological analysis cannot be answered within the framework of this observational study, given the fact that the results of the NGS analysis were not immediately available and therefore could not influence the patients'treatment. Thus, further prospective studies with higher levels of evidence are needed to investigate whether gene-based microbiological analysis will improve the treatment outcomes of patients with odontogenic abscesses.

## Conclusions

This study highlights the potential advantages of NGS in the microbiological diagnosis of odontogenic abscesses. Our findings demonstrate that gene-based microbiological analysis is superior to conventional methods, particularly in detecting anaerobic bacteria and antibiotic resistance. The ability of NGS to identify pathogens and resistance genes independent of bacterial cultivation addresses a critical limitation of traditional diagnostics, which often fail to detect anaerobes due to oxygen exposure during sample processing.

Despite these advantages, our study did not establish a direct impact of NGS on clinical outcomes, such as the LOS, primarily due to the delayed availability of sequencing results. This underscores the importance of integrating NGS into real-time clinical workflows to assess its potential benefits in optimizing targeted antibiotic therapy. Our findings reinforce that early, appropriate antibiotic therapy significantly influences patient outcomes, with resistant infections leading to prolonged hospitalisation due to delays in adjusting treatment regimens.

The integration of rapid gene-based diagnostics into routine clinical practice could therefore enhance the management of odontogenic infections by enabling more precise and timely antibiotic stewardship.

## Data Availability

Data is contained within the article.
